# On Certain Problems of Vertebrate Embryology

**Published:** 1899-06

**Authors:** 


**On Certain Problems of Vertebrate Embryology.** By John Beard, D.Sc. Pp. vii., 77. Jena: Gustav Fischer. 1896.

**The Span of Gestation and the Cause of Birth.** By John Beard, D.Sc. Pp. ix., 132. Jena: Gustav Fischer. 1897.

The author, in these two essays, puts forward a theory to explain certain phenomena which occur in the process of development of vertebrates. The theory is, that there is a hidden alternation of generations—an asexual larval form, succeeded by a sexual one. To the first belong such structures as a yolk-sac, transient nervous system and notochord, which in all vertebrate groups are found to disappear together and at a period when the embryo is just beginning to assume the body form characteristic of the adult. He calls this time the "critical period," and the interval from fertilisation of the ovum to this a "critical unit."

Monotremes alone of mammals have a well-filled yolk-sac, which is empty in all higher mammals. The cause of this disappearance of the yolk is probably to be found in the development of mammæ so that the young can be born in an early stage, the "critical" one, *i.e.* as soon as they are able to suck. This actually occurs in marsupials, and in such forms no allantoic placenta is ever formed. Most mammals, however, have a longer gestation-period, which becomes possible owing to the development of an allantoic placenta, and in such forms the gestation period is found to be a multiple of the "critical unit;" *e.g.* in the rabbit it is two, in the cow five, and in man six. The development of allantoic placenta renders the development of a marsupial pouch unnecessary.

In the case of some mammals, *e.g.* rabbit, the adult female is always with young, parturition is followed immediately by fertilisation; ovulation succeeds immediately on parturition. In other mammals, *e.g.* pig, lactation abolishes ovulation during its continuance.

The menstrual flow is not equivalent to the period of heat in lower animals, does not correspond with ovulation ; but is to be regarded as the shedding and abortion of a decidua prepared for an egg given off at the close of the preceding menstruation, but which was not fertilised. The ovulation-cycle is equal in length to the menstrual cycle; but the time of ovulation does not correspond with that of the catamenial flow.

The length of the "critical unit" in man is found to be from 45—47 days, *i.e.* twice the ovulation period. Abortions are particularly liable to occur at the end of the first "critical unit," which, according to the theory, represents an important change in the life-history of the individual, that in which the alternation of generations takes place, and at which the allantoic placenta is first formed, nourishment previously to this being obtained through the ecto-placenta derived from the yolk-sac.

The cause of birth is to be sought in a coming ovulation, *i.e.* is due reflexly to the ovary, and the formation of a corpus luteum is to be regarded as a means whereby ovulation is prevented during gestation.

The theory, of which the above is the slightest possible sketch, thus furnishes a clue to many normal and abnormal phenomena of the development of the embryo, and to the various periods at which they do or may occur. Whether it will be substantiated by future research remains to be seen.

